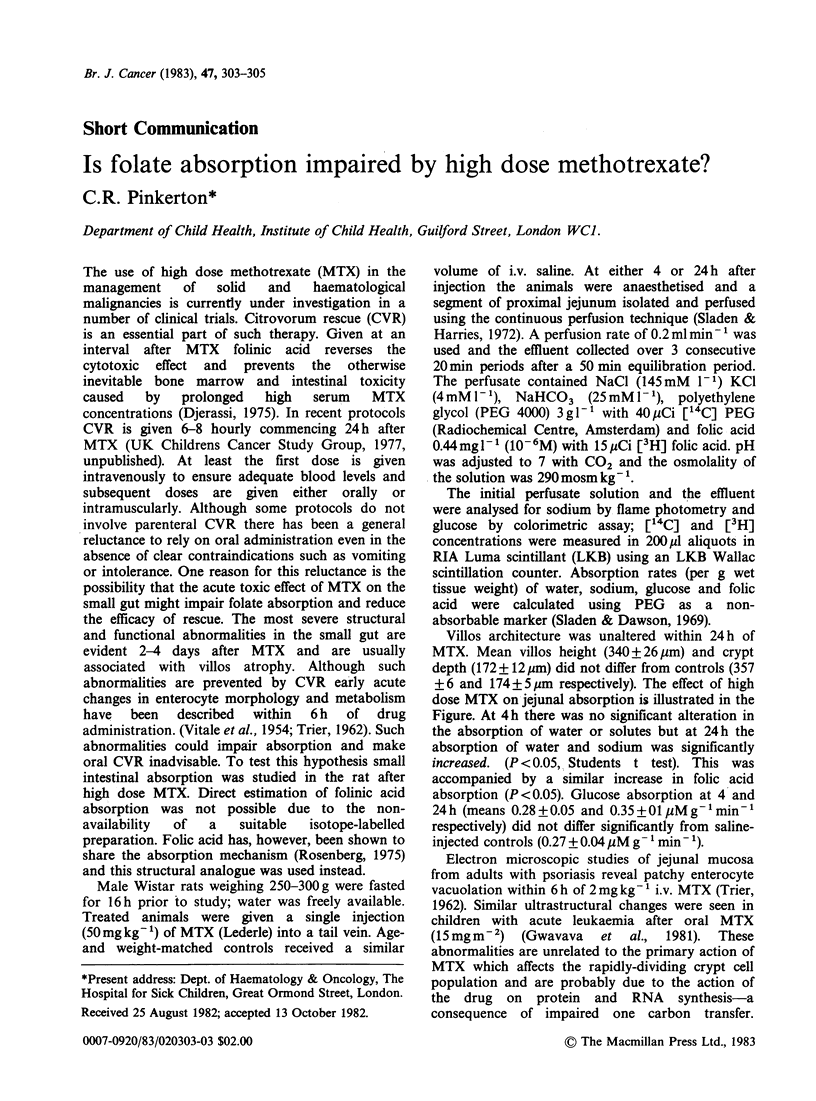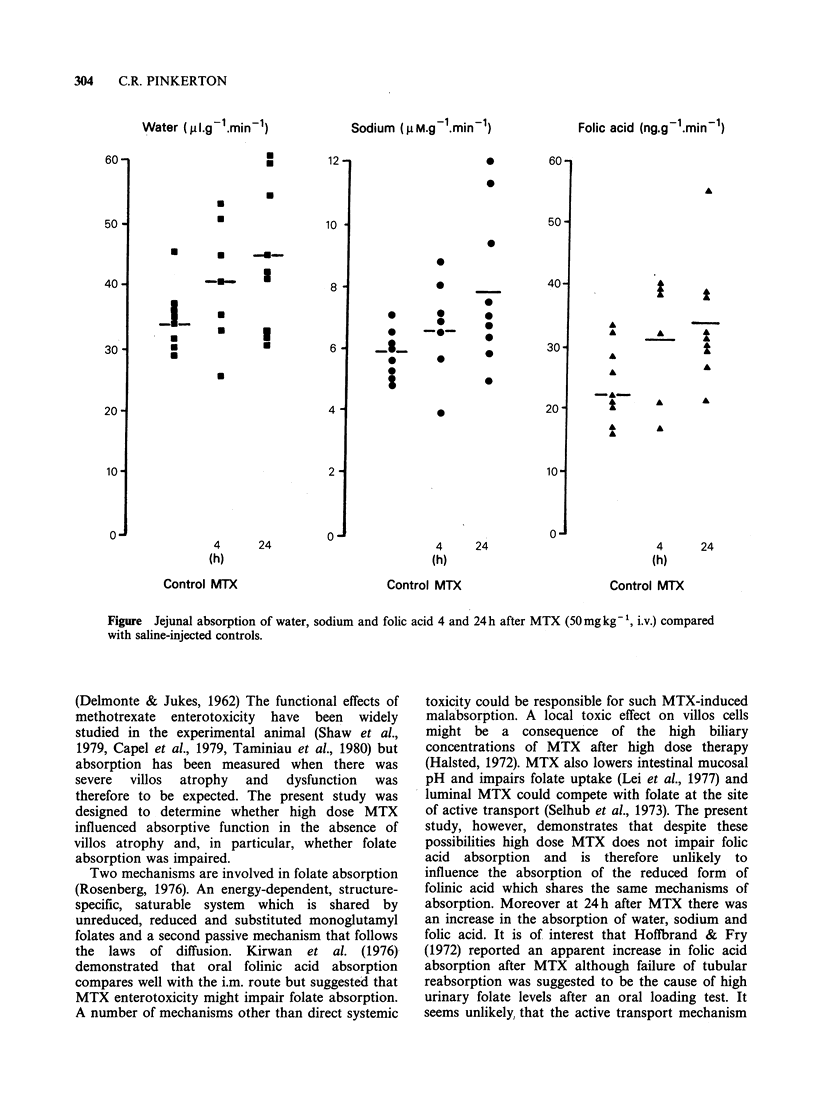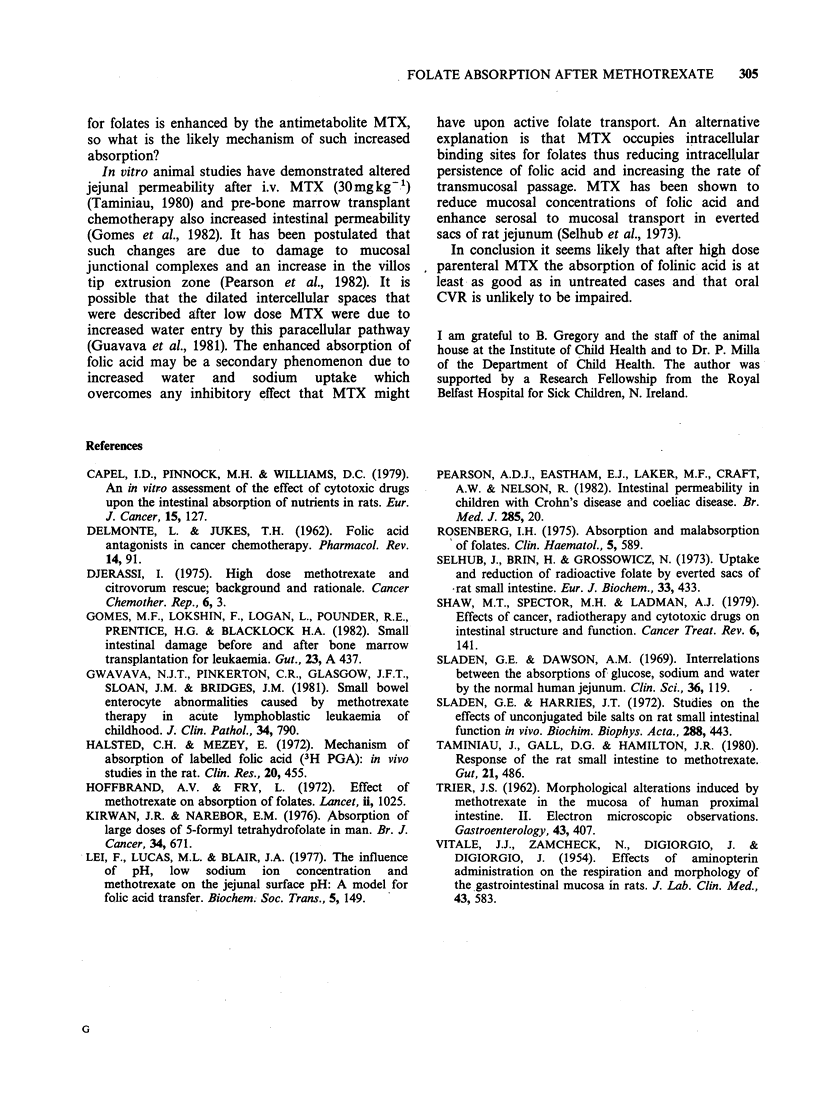# Is folate absorption impaired by high dose methotrexate?

**DOI:** 10.1038/bjc.1983.40

**Published:** 1983-02

**Authors:** C. R. Pinkerton


Br. J. Cancer (1983), 47, 303–305

**Short Communication**

### Is folate absorption impaired by high dose methotrexate?

C.R. Pinkerton*

*Department of Child Health, Institute of Child Health, Guilford Street, London WC1.*

The use of high dose methotrexate (MTX) in the management of solid and haematological malignancies is currently under investigation in a number of clinical trials. Citrovorum rescue (CVR) is an essential part of such therapy. Given at an interval after MTX folinic acid reverses the cytotoxic effect and prevents the otherwise inevitable bone marrow and intestinal toxicity caused by prolonged high serum MTX concentrations (Djerassi, 1975). In recent protocols CVR is given 6–8 hourly commencing 24h after MTX (UK Childrens Cancer Study Group, 1977, unpublished). At least the first dose is given intravenously to ensure adequate blood levels and subsequent doses are given either orally or intramuscularly. Although some protocols do not involve parenteral CVR there has been a general reluctance to rely on oral administration even in the absence of clear contraindications such as vomiting or intolerance. One reason for this reluctance is the possibility that the acute toxic effect of MTX on the small gut might impair folate absorption and reduce the efficacy of rescue. The most severe structural and functional abnormalities in the small gut are evident 2–4 days after MTX and are usually associated with villos atrophy. Although such abnormalities are prevented by CVR early acute changes in enterocyte morphology and metabolism have been described within 6h of drug administration. (Vitale *et al.*, 1954; Trier, 1962). Such abnormalities could impair absorption and make oral CVR inadvisable. To test this hypothesis small intestinal absorption was studied in the rat after high dose MTX. Direct estimation of folinic acid absorption was not possible due to the non-availability of a suitable isotope-labelled preparation. Folic acid has, however, been shown to share the absorption mechanism (Rosenberg, 1975) and this structural analogue was used instead.

Male Wistar rats weighing 250–300g were fasted for 16h prior to study; water was freely available. Treated animals were given a single injection ($50\,mg\,kg^{-1}$) of MTX (Lederle) into a tail vein. Age- and weight-matched controls received a similar volume of i.v. saline. At either 4 or 24h after injection the animals were anaesthetised and a segment of proximal jejunum isolated and perfused using the continuous perfusion technique (Sladen & Harries, 1972). A perfusion rate of $0.2\,ml\,min^{-1}$ was used and the effluent collected over 3 consecutive 20min periods after a 50 min equilibration period. The perfusate contained NaCl ($145\,mM\,l^{-1}$) KCl ($4\,mM\,l^{-1}$), $NaHCO_3$ ($25\,mM\,l^{-1}$), polyethylene glycol (PEG 4000) $3\,g\,l^{-1}$ with $40\,\mu Ci$ [$^{14}C$] PEG (Radiochemical Centre, Amsterdam) and folic acid $0.44\,mg\,l^{-1}$ ($10^{-6}M$) with $15\,\mu Ci$ [$^{3}H$] folic acid. pH was adjusted to 7 with $CO_2$ and the osmolality of the solution was $290\,mosm\,kg^{-1}$.

The initial perfusate solution and the effluent were analysed for sodium by flame photometry and glucose by colorimetric assay; [$^{14}C$] and [$^{3}H$] concentrations were measured in $200\,\mu l$ aliquots in RIA Luma scintillant (LKB) using an LKB Wallac scintillation counter. Absorption rates (per g wet tissue weight) of water, sodium, glucose and folic acid were calculated using PEG as a non-absorbable marker (Sladen & Dawson, 1969).

Villos architecture was unaltered within 24h of MTX. Mean villos height ($340 \pm 26\,\mu m$) and crypt depth ($172 \pm 12\,\mu m$) did not differ from controls (357 $\pm 6$ and $174 \pm 5\,\mu m$ respectively). The effect of high dose MTX on jejunal absorption is illustrated in the Figure. At 4h there was no significant alteration in the absorption of water or solutes but at 24h the absorption of water and sodium was significantly *increased.* ($P<0.05$, Students t test). This was accompanied by a similar increase in folic acid absorption ($P<0.05$). Glucose absorption at 4 and 24h (means $0.28 \pm 0.05$ and $0.35 \pm 01\,\mu M\,g^{-1}\,min^{-1}$ respectively) did not differ significantly from saline-injected controls ($0.27 \pm 0.04\,\mu M\,g^{-1}\,min^{-1}$).

Electron microscopic studies of jejunal mucosa from adults with psoriasis reveal patchy enterocyte vacuolation within 6h of $2\,mg\,kg^{-1}$ i.v. MTX (Trier, 1962). Similar ultrastructural changes were seen in children with acute leukaemia after oral MTX ($15\,mg\,m^{-2}$) (Gwavava *et al.*, 1981). These abnormalities are unrelated to the primary action of MTX which affects the rapidly-dividing crypt cell population and are probably due to the action of the drug on protein and RNA synthesis—a consequence of impaired one carbon transfer.

*Present address: Dept. of Haematology & Oncology, The Hospital for Sick Children, Great Ormond Street, London.

Received 25 August 1982; accepted 13 October 1982.

0007-0920/83/020303-03 $02.00

© The Macmillan Press Ltd., 1983

**Figure** Jejunal absorption of water, sodium and folic acid 4 and 24 h after MTX (50 mg kg$^{-1}$, i.v.) compared with saline-injected controls.

(Delmonte & Jukes, 1962) The functional effects of methotrexate enterotoxicity have been widely studied in the experimental animal (Shaw *et al.*, 1979, Capel *et al.*, 1979, Taminiau *et al.*, 1980) but absorption has been measured when there was severe villos atrophy and dysfunction was therefore to be expected. The present study was designed to determine whether high dose MTX influenced absorptive function in the absence of villos atrophy and, in particular, whether folate absorption was impaired.

Two mechanisms are involved in folate absorption (Rosenberg, 1976). An energy-dependent, structure-specific, saturable system which is shared by unreduced, reduced and substituted monoglutamyl folates and a second passive mechanism that follows the laws of diffusion. Kirwan *et al.* (1976) demonstrated that oral folinic acid absorption compares well with the i.m. route but suggested that MTX enterotoxicity might impair folate absorption. A number of mechanisms other than direct systemic toxicity could be responsible for such MTX-induced malabsorption. A local toxic effect on villos cells might be a consequence of the high biliary concentrations of MTX after high dose therapy (Halsted, 1972). MTX also lowers intestinal mucosal pH and impairs folate uptake (Lei *et al.*, 1977) and luminal MTX could compete with folate at the site of active transport (Selhub *et al.*, 1973). The present study, however, demonstrates that despite these possibilities high dose MTX does not impair folic acid absorption and is therefore unlikely to influence the absorption of the reduced form of folinic acid which shares the same mechanisms of absorption. Moreover at 24 h after MTX there was an increase in the absorption of water, sodium and folic acid. It is of interest that Hoffbrand & Fry (1972) reported an apparent increase in folic acid absorption after MTX although failure of tubular reabsorption was suggested to be the cause of high urinary folate levels after an oral loading test. It seems unlikely, that the active transport mechanism for folates is enhanced by the antimetabolite MTX, so what is the likely mechanism of such increased absorption?

*In vitro* animal studies have demonstrated altered jejunal permeability after i.v. MTX ($30 \text{mg kg}^{-1}$) (Taminiau, 1980) and pre-bone marrow transplant chemotherapy also increased intestinal permeability (Gomes *et al.*, 1982). It has been postulated that such changes are due to damage to mucosal junctional complexes and an increase in the villos tip extrusion zone (Pearson *et al.*, 1982). It is possible that the dilated intercellular spaces that were described after low dose MTX were due to increased water entry by this paracellular pathway (Guavava *et al.*, 1981). The enhanced absorption of folic acid may be a secondary phenomenon due to increased water and sodium uptake which overcomes any inhibitory effect that MTX might have upon active folate transport. An alternative explanation is that MTX occupies intracellular binding sites for folates thus reducing intracellular persistence of folic acid and increasing the rate of transmucosal passage. MTX has been shown to reduce mucosal concentrations of folic acid and enhance serosal to mucosal transport in everted sacs of rat jejunum (Selhub *et al.*, 1973).

In conclusion it seems likely that after high dose parenteral MTX the absorption of folinic acid is at least as good as in untreated cases and that oral CVR is unlikely to be impaired.

I am grateful to B. Gregory and the staff of the animal house at the Institute of Child Health and to Dr. P. Milla of the Department of Child Health. The author was supported by a Research Fellowship from the Royal Belfast Hospital for Sick Children, N. Ireland.

**References**

CAPEL, I.D., PINNOCK, M.H. & WILLIAMS, D.C. (1979). An *in vitro* assessment of the effect of cytotoxic drugs upon the intestinal absorption of nutrients in rats. *Eur. J. Cancer,* **15,** 127.

DELMONTE, L. & JUKES, T.H. (1962). Folic acid antagonists in cancer chemotherapy. *Pharmacol. Rev.* **14,** 91.

DJERASSI, I. (1975). High dose methotrexate and citrovorum rescue; background and rationale. *Cancer Chemother. Rep.,* **6,** 3.

GOMES, M.F., LOKSHIN, F., LOGAN, L., POUNDER, R.E., PRENTICE, H.G. & BLACKLOCK H.A. (1982). Small intestinal damage before and after bone marrow transplantation for leukaemia. *Gut.,* **23,** A 437.

GWAVAVA, N.J.T., PINKERTON, C.R., GLASGOW, J.F.T., SLOAN, J.M. & BRIDGES, J.M. (1981). Small bowel enterocyte abnormalities caused by methotrexate therapy in acute lymphoblastic leukaemia of childhood. *J. Clin. Pathol.,* **34,** 790.

HALSTED, C.H. & MEZEY, E. (1972). Mechanism of absorption of labelled folic acid ($^3$H PGA): *in vivo* studies in the rat. *Clin. Res.,* **20,** 455.

HOFFBRAND, A.V. & FRY, L. (1972). Effect of methotrexate on absorption of folates. *Lancet,* **ii,** 1025.

KIRWAN, J.R. & NAREBOR, E.M. (1976). Absorption of large doses of 5-formyl tetrahydrofolate in man. *Br. J. Cancer,* **34,** 671.

LEI, F., LUCAS, M.L. & BLAIR, J.A. (1977). The influence of pH, low sodium ion concentration and methotrexate on the jejunal surface pH: A model for folic acid transfer. *Biochem. Soc. Trans.,* **5,** 149.

PEARSON, A.D.J., EASTHAM, E.J., LAKER, M.F., CRAFT, A.W. & NELSON, R. (1982). Intestinal permeability in children with Crohn's disease and coeliac disease. *Br. Med. J.* **285,** 20.

ROSENBERG, I.H. (1975). Absorption and malabsorption of folates. *Clin. Haematol.,* **5,** 589.

SELHUB, J., BRIN, H. & GROSSOWICZ, N. (1973). Uptake and reduction of radioactive folate by everted sacs of rat small intestine. *Eur. J. Biochem.,* **33,** 433.

SHAW, M.T., SPECTOR, M.H. & LADMAN, A.J. (1979). Effects of cancer, radiotherapy and cytotoxic drugs on intestinal structure and function. *Cancer Treat. Rev.* **6,** 141.

SLADEN, G.E. & DAWSON, A.M. (1969). Interrelations between the absorptions of glucose, sodium and water by the normal human jejunum. *Clin. Sci.,* **36,** 119.

SLADEN, G.E. & HARRIES, J.T. (1972). Studies on the effects of unconjugated bile salts on rat small intestinal function *in vivo. Biochim. Biophys. Acta.,* **288,** 443.

TAMINIAU, J., GALL, D.G. & HAMILTON, J.R. (1980). Response of the rat small intestine to methotrexate. *Gut,* **21,** 486.

TRIER, J.S. (1962). Morphological alterations induced by methotrexate in the mucosa of human proximal intestine. II. Electron microscopic observations. *Gastroenterology,* **43,** 407.

VITALE, J.J., ZAMCHECK, N., DIGIORGIO, J. & DIGIORGIO, J. (1954). Effects of aminopterin administration on the respiration and morphology of the gastrointestinal mucosa in rats. *J. Lab. Clin. Med.,* **43,** 583.